# Antimicrobial Peptides: Classification, Design, Application and Research Progress in Multiple Fields

**DOI:** 10.3389/fmicb.2020.582779

**Published:** 2020-10-16

**Authors:** Yuchen Huan, Qing Kong, Haijin Mou, Huaxi Yi

**Affiliations:** College of Food Science and Engineering, Ocean University of China, Qingdao, China

**Keywords:** antimicrobial peptides, classification, coronavirus, mode of action, design, motifs, application

## Abstract

Antimicrobial peptides (AMPs) are a class of small peptides that widely exist in nature and they are an important part of the innate immune system of different organisms. AMPs have a wide range of inhibitory effects against bacteria, fungi, parasites and viruses. The emergence of antibiotic-resistant microorganisms and the increasing of concerns about the use of antibiotics resulted in the development of AMPs, which have a good application prospect in medicine, food, animal husbandry, agriculture and aquaculture. This review introduces the progress of research on AMPs comprehensively and systematically, including their classification, mechanism of action, design methods, environmental factors affecting their activity, application status, prospects in various fields and problems to be solved. The research progress on antivirus peptides, especially anti-coronavirus (COVID-19) peptides, has been introduced given the COVID-19 pandemic worldwide in 2020.

## Introduction

Alexander Fleming discovered lysozyme in 1922, and this discovery marked the birth of modern innate immunity. Since then, antibiotics and antimicrobial peptides (AMPs) have been discovered. A total of 3,240 AMPs have been reported in the antimicrobial peptide database (APD3^1^) updated on August 24, 2020.

Different types of AMPs have the following commonalities: their number of amino acid residues is between 10 and 60 (average: 33.26), and almost all AMPS are cationic (average net charge: 3.32). However, several anionic AMPs also exist, and they have several acidic amino acids like aspartic acid and glutamic acid (Malkoski et al., 2001; Schittek et al., 2001; Lai et al., 2007).

The anti-microbial resistance of microorganisms is becoming increasingly serious with the abuse of antibiotics in medicine, agriculture and animal husbandry, especially in developing countries. Research from Kenya has detected substantial amounts of antibiotic residues in edible meat (Ayukekbong et al., 2017). The prevalence of vancomycin-resistant *Enterococcus* (VRE) and methicillin-resistant *Staphylococcus aureus* (MRSA) is increasing in clinical medicine, so the countermeasures are urgently needed to address these bacterial infections. However, from the perspective of pharmaceutical companies, the development of new antibiotic drugs results in low profit. Thus, replacing antibiotics has become a consideration in the pharmaceutical, agricultural, animal husbandry, and food industries.

Research on AMPs is continuously developing and considerable amounts of data on AMPs have been stored in AMP databases. However, the mechanism of AMPs remains incompletely understood, and further work needs to be performed to determine the relationship between different physicochemical properties to obtain low-cost and highly safe AMPs with remarkable antimicrobial effects and the specificity and a high capacity for synergies of AMPs should also be further developed (Lazzaro et al., 2020).

## Classification of AMPs

**FIGURE 1:**
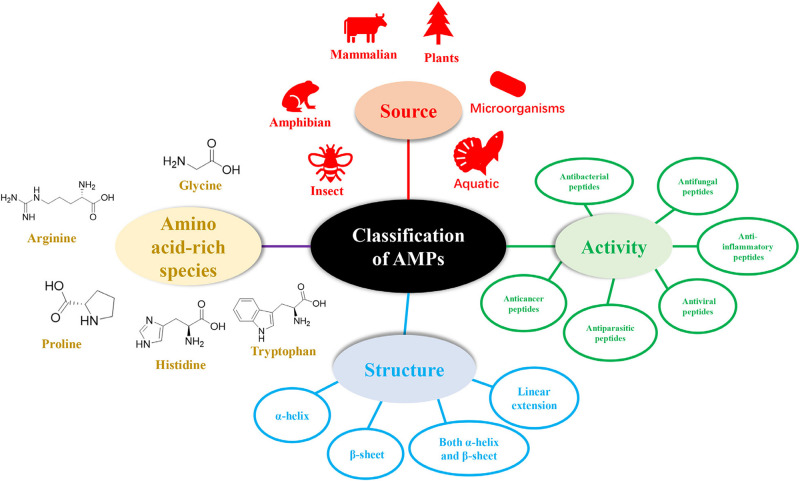
Classification of antimicrobial peptides.

The diversity of natural AMPs causes difficulty in their classification. AMPs are classified based on (1) source, (2) activity, (3) structural characteristics, and (4) amino acid-rich species (Figure 1).

### Classification of AMPs Based on Sources

The sources of AMPs can be divided into mammals (human host defense peptides account for a large proportion), amphibians, microorganisms, and insects according to statistical data in APD3. The AMPs found in oceans have also attracted widespread attention.

#### Mammalian Antimicrobial Peptides

Mammalian antimicrobial peptides are found in human, sheep, cattle, and other vertebrates. Cathelicidins and defensins are the main families of AMPs. Defensins can be divided into α-, β-, and θ-defensins depending on the position of disulfide bonds (Reddy et al., 2004). Human host defense peptides (HDPs) can protect human from microbial infections but show different expressions in every stage of human growth. For example, cathelicidin LL-37, a famous AMP derived from the human body, is usually detected in the skin of newborn infants, whereas human beta-defensin 2 (hBD-2) is often expressed in the elderly instead of the young (Gschwandtner et al., 2014). HDPs can be identified in many parts of the body such as skin, eyes, ears, mouth, respiratory tract, lung, intestine, and urethra. Besides, AMPs in human breast milk also play an important role in breastfeeding because it can decrease the morbidity and mortality of breast-feeding infants (Field, 2005). What’s interesting is that Casein201 (peptide derived from β-Casein 201–220 aa), identified in colostrum, shows different levels in preterm human colostrum and term human colostrums (Zhang et al., 2017). Dairy is an important source of AMPs, which are generated through milk enzymatic hydrolysis. Several AMPs have been identified from α-lactalbumin, β-lactoglobulin, lactoferrin, and casein fractions, and the most famous peptide obtained is lactoferricin B (LfcinB) (Sibel Akalın, 2014). Furthermore, whether the AMPs derived from dairy products can be used for dairy preservation is also an interesting subject to develop.

In addition to antimicrobial activity, HDPs, such as cathelicidins and defensins, also affect immune regulation, apoptosis, and wound healing (Wang, 2014).

#### Amphibian-Derived Antimicrobial Peptides

Antimicrobial peptides from amphibians play an important role in the protection of amphibians from the pathogens that have induced the global amphibian population decline (Rollins-Smith, 2009). Frogs are the main source of amphibian AMPs and the most famous AMP from frogs is magainin; the skin secretions of frogs from genera *Xenopus*, *Silurana*, *Hymenochirus*, and *Pseudhymenochirus* under the Pipidae family are rich in AMPs (Conlon and Mechkarska, 2014). Furthermore, cancrin, which has an amino acid sequence of GSAQPYKQLHKVVNWDPYG, has been reported as the first AMP from the sea amphibian *Rana cancrivora* (Lu et al., 2008). This marks a broader source of AMPs of amphibians.

#### Insect-Derived Antimicrobial Peptides

Antimicrobial peptides are mainly synthesized in fat bodies and blood cells of insects, which is one of the main reasons for insects’ strong adaptability to survival (Vilcinskas, 2013). Cecropin is the most famous family of AMPs from insects, and it can be found in guppy silkworm, bees, *Drosophila*. Cecropin A shows activity against different inflammatory diseases and cancers (Dutta et al., 2019). What should be known is that the number of AMPs varies greatly between species, for example, invasive harlequin ladybird (*Harmonia axyridis*) and black soldier fly (*Hermetia illucens*) have up to 50 AMPs, while pea aphid (*Acyrthosiphon pisum*) lacks AMPs (Shelomi et al., 2020). Jellein, a peptide derived from bee royal jelly, shows promising effects on several bacteria and fungi, and its lauric acid-conjugated form can inhibit the parasite *Leishmania major* (Zahedifard et al., 2020).

#### Microorganisms-Derived Antimicrobial Peptides

Antimicrobial peptides can be obtained from microorganisms like bacteria and fungi, and some famous peptides are nisin, gramicidin from *Lactococcus lactis*, *Bacillus subtilis*, and *Bacillus brevis* (Cao et al., 2018). Due to the high price of chemical synthesis of AMPs, the biological expression has attracted the increase of attention. Specific yeast species like *Pichia pastoris*, *Saccharomyces cerevisiae*, and bacteria like *Escherichia coli*, *B. subtilis*, and plants have been used for expression systems (Parachin et al., 2012), but it should be noticed that because of the toxicity, proteolytic degradation, and purification, AMPs are difficult to be produced in *E. coli*, which is necessary to take advantage of fusion tags (Yu et al., 2015).

Besides, numerous AMPs have also been extracted and isolated from the stems, seeds, and leaves of plants, and they are classified into several groups, including thionins, defensins and snakins (Tang et al., 2018). More marine-derived AMPs have been reported to have given the increasing value allotted by people to marine resources. Although most of the reported marine AMPs have been tested *in vitro*, several of these AMPs have shown promising results *in vivo*, for example, As-CATH4 shows an immunity-stimulating effect *in vivo* and can enhance the anti-infective capability of drugs used in combination with it (Semreen et al., 2018). Myticusin-beta is an immune-related AMP of *Mytilus coruscus* and a promising alternative to antibiotics (Oh et al., 2020). Moreover, GE33, known as pardaxin, is a marine AMP and the GE33-based vaccine has shown the ability to enhance antitumor immunity in mice (Huang et al., 2013).

### Classification Based on Activity

**FIGURE 2:**
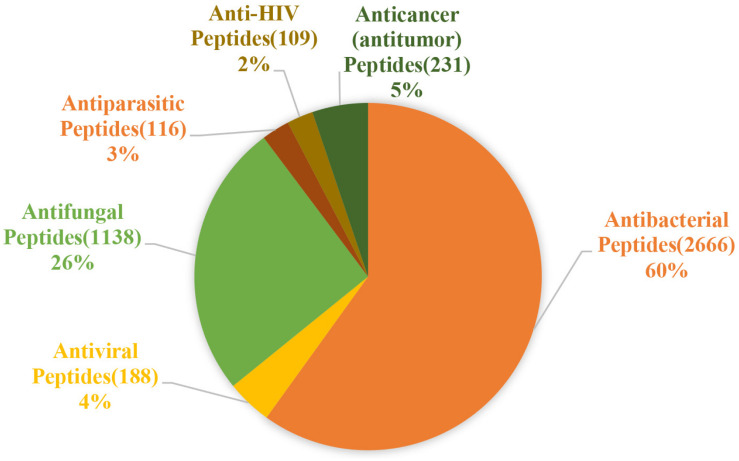
Statistics of the main functions of antimicrobial peptides. Antibacterial peptides account for the largest proportion, approximately 60%, followed by antifungal peptides, which account for 26%, and antiviral, antiparasitic, anticancer, anti-HIV peptides account for almost the same about 2–5% (the figure is drawn based on data in APD3).

The activity of AMPs can be divided into 18 categories according to the statistics of the ADP3 database. These categories can be summarized as antibacterial, antiviral, antifungal, antiparasitic, anti-human immunodeficiency virus (HIV), and anti-tumor peptides (Figure 2).

#### Antibacterial Peptides

Antibacterial peptides account for a large part of AMPs and have a broad inhibitory effect on common pathogenic bacteria, such as VRE, *Acinetobacter baumannii*, and MRSA in clinical medicine and *S. aureus*, *Listeria monocytogenes*, *E. coli* in food and *Salmonella*, *Vibrio parahaemolyticus* in aquatic products. Many natural and synthetic AMPs like nisin, cecropins and defensins have shown good inhibition activity to Gram-positive bacteria and Gram-negative bacteria. In recent research, AMPs P5 (YIRKIRRFFKKLKKILKK-NH_2_) and P9 (SYERKINRHFKTLKKNLKKK-NH_2_), which are designed based on *Aristicluthys nobilia* interferon-I, inhibit MRSA and show a low cytotoxicity (Li C. et al., 2019).

#### Antifungal Peptides (AFPs)

Antifungal peptides are a subclass of AMPs that address fungal infections with enhanced drug resistance. Many AFPs have shown excellent anti-fungal activities against common pathogenic fungi, such as *Aspergillus* and *Candida albicans* in clinical medicine, yeast, filamentous fungi (e.g., *Aspergillus flavus*), mold in food and agriculture. Except for brevinin, ranatuerin, cecropins, many synthetic peptides also show good antifungal activity. For example, AurH1, derived from aurein 1.2, can effectively treat *C. albicans* infection, which has a lethal rate up to 40% (Madanchi et al., 2020). Aflatoxin, which is a carcinogen produced by *A. flavus*, is harmful to the human body. Many AFPs can inhibit the growth of *A. flavus*. For example, an AFP with a sequence of FPSHTGMSVPPP can inhibit the growth of *A. flavus* MD3. A total of 37 antifungal peptides isolated from *Lactobacillus plantarum* TE10 and their mixture can reduce *A. flavus* spore formation in fresh maize seeds (Muhialdin et al., 2020). Moreover, two chemically synthesized radish AMPs show a good inhibitory effect against different yeast species, such as *Zygosaccharomyces bailii* and *Zygosaccharomyces rouxii* (Shwaiki et al., 2020).

#### Antiviral Peptides (AVPs)

**FIGURE 3:**
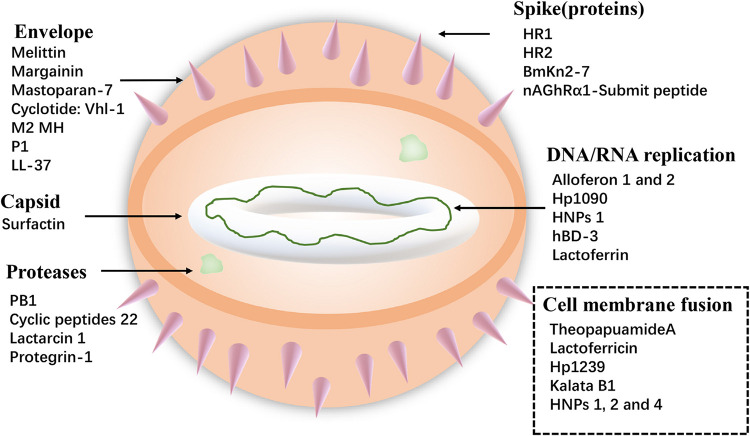
Examples of specific targets for Antiviral peptides.

Viruses cause serious harm to human life and huge economic losses to the animal husbandry. The COVID-19, which is the recent outbreak, has caused great loss of lives and properties. Furthermore, foot-and-mouth disease virus, avian influenza virus (AIV), and HIV are long-term threats to human life. So, it is extremely urgent to solve these problems, and antiviral peptides provide new ways. Antiviral peptides show a strong killing effect on viruses mainly by (1) inhibiting virus attachment and virus cell membrane fusion, (2) destroying the virus envelope, or (3) inhibiting virus replication (Jung et al., 2019) (shown in Figure 3). A recent report has shown that AMP Epi-1 mediates the inactivation of virus particles and has good inhibitory activity against foot-and-mouth disease virus (Huang et al., 2018). Moreover, infectious bronchitis virus (IBV) is the pathogen of infectious bronchitis and the inoculation of swine intestinal AMP (SIAMP)–IBV mixed solution remarkably reduced the mortality of chicken embryos compared with the IBV infection group, showing the good inhibitory activity of SIAMP on IBV (Sun et al., 2010). Anti-HIV peptides are a subclass of anti-viral peptides. The most important examples of these peptides include defensins (including α- and β-defensins, which have different mechanisms), LL-37, gramicidin D, caerin 1, maximin 3, magainin 2, dermaseptin-S1, dermaseptin-S4, siamycin-I, siamycin-II, and RP 71955 (Madanchi et al., 2020) and antiviral peptide Fuzeon^TM^ (enfuvirtide) has been commercialized as an anti-HIV drug (Ashkenazi et al., 2011).

**FIGURE 4:**
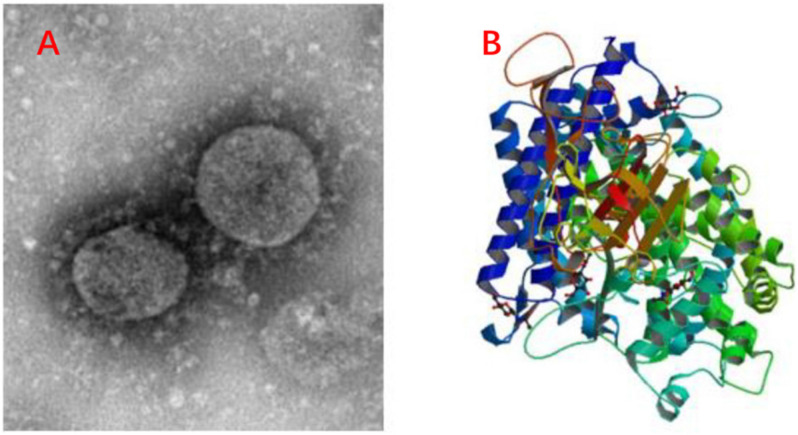
Information of COVID-19. **(A)** C-F13-nCoV Wuhan strain 02, Strain Number: CHPC 2020.00002; NPRC 2020.00002, Source: National Pathogen Resource Collection Center (National Institute for Viral Disease Control and Prevention under Chinese Center for Disease Control and Prevention). **(B)** Structure of novel coronavirus spike receptor-binding domain complexed with its receptor ACE2. (10.2210/pdb6LZG/pdb).

Due to the global spread of the COVID-19 (Figure 4A), the antiviral peptides against the coronavirus will be discussed in more detail. Coronaviruses (CoVs) belong to the family Coronaviridae; they are enveloped viruses with a positive-sense single-stranded RNA genome and have a helical symmetry (Franks and Galvin, 2014). CoVs, including severe acute respiratory syndrome CoV (SARS-CoV) and Middle East respiratory syndrome coronavirus (MERS-CoV) (Mustafa et al., 2018), and the recent outbreak of COVID-19 have caused serious threats to human life and property. CoVs can cause life-threatening respiratory diseases and the viral particle is formed by spike glycoprotein (S), the envelope (E), the membrane (M), and the nucleocapsid (N) (Vilas Boas et al., 2019). It should be noted that their infectivity requires viral spike (S) protein. Fusion inhibitor peptides combine with the S protein to interfere with its folding and prevent infection. Besides, the S2 domain of the SARS-CoV S protein contains heptad repeat HR1 and HR2 sequences. Peptide HR2 (HR2: SLTQINTTLLDLTYEMLSLQQVVKALNESYIDLKEL) and its lipid-binding peptide is highly similar or even identical to the near-membrane portion of S protein ferredoxin, which interferes with refolding into post-fusion fusion-catalyzing domains (FDs) (Du et al., 2009; Park and Gallagher, 2017). According to recent research, the lipopeptide EK1C4, derived from EK1 (SLDQINVTFLDLEYEMKKLEEAIKKLEESYIDLKEL), is the most effective fusion inhibitor against COVID-19 S protein-mediated membrane fusion (Xia et al., 2020). Homology modeling and protein-peptide docking showed that temporin has potential therapeutic applications against MERS-CoV (Marimuthu et al., 2019). Two AMPs from the non-structural protein nsp10 of SARS-CoV, K12, and K29, can inhibit SARS-CoV replication (Ke et al., 2012). Furthermore, rhesus theta-defensin 1 (RTD-1) treated animals have a marked reduction in mortality in the presence of SARS-CoV while the peptide alone shows airway inflammation and the one possible mechanism of action for RTD-1 is immunomodulatory (Wohlford-Lenane et al., 2009). In general, AMPs against coronavirus can be roughly classified as i) peptides derived from HR1, HR2 and RBD subunits of the spike protein, ii) peptides derived from other AMPs, iii) Peptides derived from non-structural protein (Mustafa et al., 2018). Furthermore, molecular docking analysis indicated that peptides were employed to disrupt the interaction between COVID-19 and ACE2 (angiotensin-converting enzyme 2) to inhibit COVID-19 entrance in cells (Figure 4B) (Souza et al., 2020). Finally, it should be noted that this therapy lacks clinical trials and the main method of animal experiments is an intranasal administration. This reminds us that nasal drug delivery (NDD) is a potential therapy for AMPs as anti-coronavirus drugs. Besides, the antiviral database AVPdb^2^ includes numerous antiviral peptides.

#### Antiparasitic Peptides

Parasitic protozoa can cause diseases in human and animals through a variety of routes, including animal-to-person or person-to-person contact, water, soil, and food (Chalmers et al., 2020). And with the increase in parasite drug resistance, the need for new treatments has increased. Antiparasitic peptides show their killing effect on parasites which cause diseases such as malaria and leishmaniasis (Mangoni et al., 2005; Rhaiem and Houimel, 2016) and AMPs like cathelicidin, temporins-SHd show high inhibition activity against parasites (Abbassi et al., 2013). In recent research, Epi-1, a marine synthetic AMP, can remarkably inhibit *Trichomonas vaginalis* by destroying its membrane (Neshani et al., 2019). The peptide Jellein derived from bee royal jelly which has introduced above and 4-amino acid AMP KDEL (lysine, aspartic acid, glutamic acid, and leucine) has shown a significant effect on the Leishmania parasite (Cao et al., 2019; Zahedifard et al., 2020). However, it should be noted that their mechanisms are not the same. Cyanobacterial peptides differ from higher-eukaryote AMPs because their antiparasitic action depends on specific protein targets. Thus, these target parasites can be distinguished accurately even though they belong to the same family or genus (Rivas and Rojas, 2019).

#### Anticancer Peptides (ACPs)

The ACPs show anticancer mechanisms by (1) recruiting immune cells (such as dendritic cells) to kill tumor cells, (2) inducing the necrosis or apoptosis of cancer cells, (3) inhibiting angiogenesis to eliminate tumor nutrition and prevent metastasis, and (4) activating certain regulatory functional proteins to interfere with the gene transcription and translation of tumor cells (Wu D. et al., 2014; Ma et al., 2020). Tritrpticin and its analogs induce considerable toxicity toward Jurkat cells *in vitro*, whereas indolicidin and puroindoline A can also act as ACPs (Arias et al., 2020). It should be noted that both net charge and hydrophobicity play important roles in optimizing the anticancer activity of ACPs and they can constrain and influence each other. Thus, achieving a balance between net charge and hydrophobicity is important for better anticancer activity.

Besides the peptide mentioned above, anti-inflammatory, anti-diabetic peptides, spermicidal peptides etc. have been noticed, but they are not the same as antimicrobial peptides. Simply put, anti-inflammatory peptides decrease the release of inflammatory mediators and inflammatory cytokines (nitric oxide, interleukin-6, and interleukin-1β) and some of them also inhibit inflammatory signals like NF-κB, MAPK, and JAK-STAT pathways (Meram and Wu, 2017; Gao et al., 2020). Anti-diabetic peptides play their function by modulating the G protein-coupled receptor kinase (GRK 2/3) or activating glucagon-like peptide-1 (GLP-1), glucagon receptors (Marya et al., 2018; Graham et al., 2020). However, it is not accurate to classify these types of peptides as AMPs and bioactive peptides may be more convincing.

### Classification of AMPs Based on Amino Acid-Rich Species

#### Proline-Rich Peptides (PrAMPs)

Proline is a typical non-polar amino acid. PrAMPs behave differently from other AMPs, that is, they enter bacterial cytoplasm by the inner membrane transporter SbmA instead of killing bacteria through membrane destruction (Mattiuzzo et al., 2007). Once in the cytoplasm, PrAMPs target ribosomes and block the binding of aminoacyl-tRNA to peptidyltransferase center or trap decoding release factors on the ribosome during the termination of translation to interfere with protein synthesis (Seefeldt et al., 2015). For instance, Tur1A, which is an orthologous AMP of bovine PrAMP Bac7 discovered from *Tursiops truncatus*, interferes with the transition from the initial phase to the extension phase of protein synthesis by binding to ribosomes. In addition, different PrAMPs lack a high sequence similarity but have short motifs containing repeating proline and arginine (Arg) residues (e.g., -PPXR- in Bac5 and -PRPX- in Bac7) (Mardirossian et al., 2018, 2019). Although PrAMPs mainly kill Gram-positive bacteria, *p*PR-AMP1, a proline-rich AMP identified from crab (*Scylla paramamosain*), exhibits antimicrobial activity against Gram-positive and Gram-negative bacteria (Imjongjirak et al., 2017). Besides, pieces of research have shown that PrAMPs have immunostimulation activity (Li W. et al., 2016).

#### Tryptophan- and Arginine-Rich Antimicrobial Peptides

Tryptophan (Trp), as a non-polar amino acid, has a remarkable effect on the interface region of the lipid bilayer, whereas Arg, as a basic amino acid, confers peptide charge and hydrogen bond interactions, which are essential properties to combine with the bacterial membrane’s abundant anionic component. And it seems that Trp residues play the role of natural aromatic activators of Arg-rich AMPs by ion-pair-π interactions (Walrant et al., 2020), thereby promoting enhanced peptide-membrane interactions (Chan et al., 2006). In addition to indolicidin and Triptrpticin which both are famous AMPs that rich in Arg and Trp residues. Octa 2 (RRWWRWWR) is also a typical Trp- and Arg-rich AMP that inhibits Gram-negative *E. coli* and *Pseudomonas aeruginosa* and Gram-positive *S. aureus*. And short Trp- and Arg-rich AMPs designed based on bovine and murine lactoferricin have also shown strong inhibitory action against bacteria (Strøm et al., 2002; Bacalum et al., 2017).

#### Histidine-Rich Peptides

Histidine is a common basic amino acid, and histidine-rich AMPs show good membrane permeation activity. HV2 is a histidine-rich AMP designed based on RR(XH)_2_XDPGX(YH)_2_RR–NH_2_ (where X represents I, W, V, and F). This peptide increases the permeability of bacterial cell membranes to cause cell membrane rupture and death. In addition, HV2 inhibits bacterial movement in a concentration-dependent manner and shows a strong anti-inflammatory effect by inhibiting the production of tumor necrosis factor α (TNF-α) (Dong et al., 2019). An AMP designed based on Octa 2 has shown good therapeutic potential by replacing its Arg residues with histidine (Bacalum et al., 2017). Furthermore, L4H4, which is designed based on the linear cationic amphiphilic peptide magainin, also shows good antibacterial activity and cell penetration properties by inserting four histidine sequences in leucine and alanine (Lointier et al., 2020).

#### Glycine-Rich Antimicrobial Peptides

The R group of glycine is generally classified as a non-polar amino acid in biology. Glycine-rich AMPs, such as attacins and diptericins, widely exist in nature (Lee et al., 2001; Kwon et al., 2008). These peptides contain 14% to 22% glycine residues, which have an important effect on the tertiary structure of the peptide chain. A glycine-rich AMP derived from salmonid cathelicidins activates phagocyte-mediated microbicidal mechanisms, which differ from the mechanism of conventional AMPs (D’Este et al., 2016). Furthermore, the glycine-rich central–symmetrical GG3 is an ideal commercial drug candidate against clinical Gram-negative bacteria (Wang et al., 2015).

### Classification Based on Antimicrobial Peptide Structures

**FIGURE 5:**
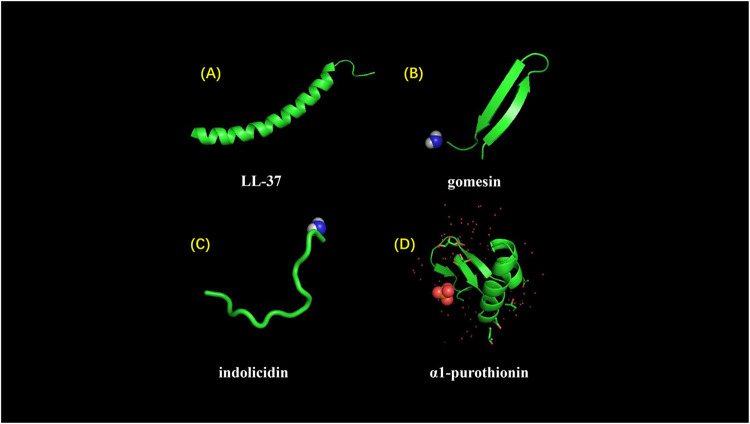
Different structures of AMPs. **(A)** LL-37 adopts a typical α-helical conformation (10.2210/pdb2K6O/pdb). **(B)** Gomesin is a β-sheet peptide and stabilized by disulfide bonds (10.2210/pdb1KFP/pdb). **(C)** Indolicidin is a AMP with linear extension structure instead of well-defined 3D structure (10.2210/pdb1G89/pdb). **(D)** α1-purothionin adopts both alpha-helix and beta-sheet conformation, and arrows indicate extension direction (10.2210/pdb2plh/pdb).

Antimicrobial peptides can be divided into four categories based on their structures including linear α-helical peptides, β-sheet peptides, linear extension structure, and both α-helix and β-sheet peptides (Figure 5) (Lei et al., 2019). Moreover, progressively cyclic peptides and AMPs with more complex topologies (including lasso peptides and thioether bridged structures) are reported (Koehbach and Craik, 2019).

## Antimicrobial Peptide Action Mechanism

### Membrane Targeting Mechanism

**FIGURE 6:**
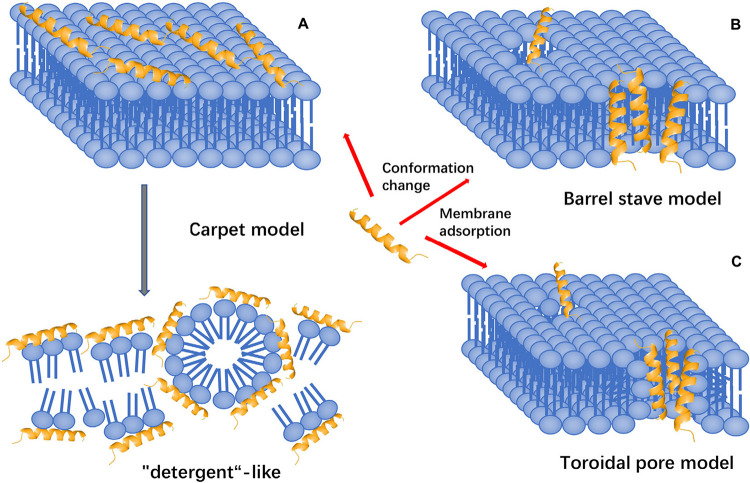
Models of action for extracellular AMP activity. **(A)** Carpet model: accumulation of AMPs on the surface and then destroy the cell membrane in the manner of “detergent”. **(B)** Barrel stave model: AMPs aggregate with each other and are inserted into the bilayer of the cell membrane in the form of multimers and arrange parallel to the phospholipids, then form a channel. **(C)** Toroidal pore model: accumulation of AMPs vertically embed in the cell membrane, and then, bend to form a ring hole.

The membrane-targeting mechanisms of AMPs can be described through models, including the pole and carpet models and the pole model can be further divided into the toroidal pore and barrel-stave models (Figure 6).

#### The Toroidal Pore Model

The toroidal pore model is also known as the wormhole model. In this model, AMPs vertically embedded in the cell membrane accumulate and then bend to form a ring hole with a diameter of 1–2 nm (Matsuzaki et al., 1995, 1996). The typical examples of this model are magainin 2, lacticin Q, and arenicin. Furthermore, cationic peptides, including TC19, TC84, and BP2, compromise the membrane barrier by creating fluid domains (Omardien et al., 2018).

#### Barrel-Stave Model

Antimicrobial peptides aggregate with each other, penetrate the bilayer of the cell membrane in the form of multimers, and form channels that result in the cytoplasmic outflow. In severe cases, AMPs can induce cell membrane collapse and lead to cell death (Lohner and Prossnigg, 2009). For instance, Alamethicin performs its pore-forming activity by using this model. Besides, hairpin AMP protegrin-1 can form stable octameric β-barrels and tetrameric arcs (half barrels) in implicit and explicit membranes by simulations (Lipkin and Lazaridis, 2015).

#### Carpet-Like Model

Antimicrobial peptides are arranged parallel to the cell membrane. Their hydrophilic end faces the solution, and their hydrophobic end faces the phospholipid bilayer. AMPs will cover the membrane surface that similar to a carpet and destroy the cell membrane in a ‘detergent’-like manner (Oren and Shai, 1998). However, this pore-forming mechanism requires a certain concentration threshold and the required concentration of AMPs is high. Human cathelicidin LL-37 exhibits its activity through this mechanism, and AMPs with β-sheet structure also play a role in this model (Shenkarev et al., 2011; Corrêa et al., 2019). Polarized light-attenuated total reflection Fourier transform infrared spectroscopy (ATR-FTIR) was used to study the effect of AMP cecropin P1 on the bacterial cell membrane and found that it was an applied flat on the surface of the pathogen’s cell membrane to destabilize and eventually destroy the cell membrane (Lyu et al., 2019).

**FIGURE 7:**
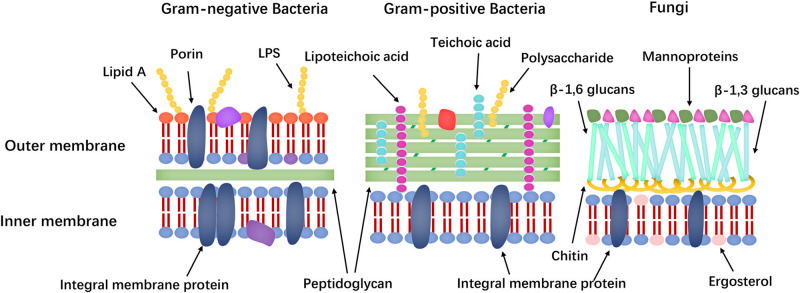
Comparison of Gram-negative bacteria, Gram-positive bacteria and fungi cell walls.

Membrane targeting mechanisms (the cell membrane composition differences of bacteria and fungi shown in Figure 7) can be further refined to address the large differences in the lipid composition of the cell membranes of bacteria, fungi, and mammals. The main lipids in cell membranes include glycerophospholipids (GPLs), lysolipids, sphingolipids, and sterols. Phosphatidylethanolamine (PE), phosphatidylglycerol (PG), and cardiolipin (CL) are the most common anionic lipids in bacteria, whereas phosphatidylcholine (PC), phosphatidylinositol (PI), PE, and phosphatidic acid (PA) are the main GPLs in fungal cell membranes (Ejsing et al., 2009; Singh and Prasad, 2011; Li et al., 2017). Furthermore, fungal cell membranes are more anionic than mammalian cell membranes and have higher PC content. Meanwhile, ergosterol is the sterol found in the plasma membrane of lower eukaryotes, such as fungi, whereas that of animals contains cholesterol (Faruck et al., 2016). Many AMPs take advantage of differences in membrane components to exert their effects.

Antimicrobial peptides are promising to be anti-biofilm agents but it should be noticed that they are different from the cell penetrating peptides (CPPs) which typically comprise 5–30 amino acids and can translocate across the cell membrane. CPPs could be categorized according to physicochemical properties into three classes: Cationic, amphipathic, and hydrophobic, but anti-biofilm peptides have stricter requirements for these physicochemical properties. Anti-biofilm peptides target the biofilms by different mechanisms including (1) degradation of signals within biofilms; (2) permeabilize within cytoplasmic membrane/EPS; (3) modulating EPS production etc. and then can address chronic multi-resistant bacterial infections (Pletzer et al., 2016; Ribeiro et al., 2016; Guidotti et al., 2017; Derakhshankhah and Jafari, 2018; Rajput and Kumar, 2018). For instance, SAAP-148, synthesized based on LL-37, showed activity to prevent biofilm formation by *S. aureus* and *A. baumannii* (Crunkhorn, 2018).

### Non-membrane Targeting Mechanism

The way of AMPs entering cells is direct penetration or endocytosis. After entering the cytoplasm, AMPs will identify and act on the target. Depending on the target, AMPs can be divided into the following categories.

#### Inhibition of Protein Biosynthesis

Antimicrobial peptides affect transcription, translation, and assembly into functional peptides through molecular chaperone folding by interfering with related enzymes and effector molecules. For example, Bac7 1–35 targets ribosomes to inhibit protein translation (Mardirossian et al., 2014), whereas Tur1A inhibits protein synthesis in *E. coli* and *Thermus thermophilus* by inhibiting the transition from the initial phase to the extension phase. However, the differences between Tur1A and Bac7 also lead to various ways of binding to ribosomes and interacting with the ribosomal peptide exit tunnel (Mardirossian et al., 2018). But some AMPs’ have different targets. For instance, genome-wide transcription shows that the AMP DM3 can affect many important intracellular pathways of protein biosynthesis (Le et al., 2016).

Chaperones are key proteins for correctly folding and assembling newly synthesized proteins and make them have stereoisomerism, which makes AMPs have cell selectivity and can prevent cytotoxicity. According to a previous review: both pyrhocoricin and drosocin can prevent DnaK from refolding misfolded proteins by inducing a permanent closure of the DnaK peptide-binding cavity (Kragol et al., 2001; Le et al., 2017; Wrońska and Boguś, 2020).

#### Inhibition of Nucleic Acid Biosynthesis

Antimicrobial peptides can affect key enzymes or induce the degradation of nucleic acid molecules to inhibit nucleic acid biosynthesis. Indolicidin, a C-terminal-amidated cationic Trp-rich AMP with 13 amino acids, specifically targets the abasic site of DNA to crosslink single- or double-stranded DNA and it can also inhibit DNA topoisomerase I (Subbalakshmi and Sitaram, 1998). TFP (Tissue factor pathway inhibitor)1-1TC24, which is an AMP from tongues, enters the cytoplasm of target cells after the rupture of cell membrane and then degrades DNA and RNA (He et al., 2017).

#### Inhibition of Protease Activity

Many AMPs can inhibit various metabolic activities by inhibiting protease activity. For example, histatin 5 has a strong inhibitory effect on the proteases secreted by the host and bacteria. AMPs eNAP-2 and indolicidin inhibit microbial serine proteases, elastase, and chymotrypsin (Le et al., 2017). Cathelicidin-BF is a peptide isolated from the venom of *Bungarus fasciatus*, it can effectively inhibit thrombin-induced platelet aggregation and further block protease-activated receptor 4 (Shu et al., 2019).

#### Inhibition of Cell Division

Antimicrobial peptides inhibit cell division by inhibiting DNA replication and DNA damage response (SOS response), blocking the cell cycle or causing the failure of chromosome separation (Lutkenhaus, 1990). For instance, APP (GLARALTRLLRQLTRQLTRA), which is an AMP with 20 amino acid residues, can efficiently kill *C. albicans* because of its cell-penetrating efficiency, strong DNA-binding affinity, and ability to induce S-phase arrest in intracellular environment (Li L. et al., 2016). MciZ, which has 40 amino acid residues, is an effective inhibitor of bacterial cell division, Z-ring formation, and localization (Cruz et al., 2020).

Moreover, it has been reported that several AFPs have damaging effects on the organelles of fungi. For example, Histintin 5 can interact with mitochondria, causing the production of ROS, and inducing cell death (Helmerhorst et al., 2001).

In addition to intracellular targets, differences in cell wall composition, such as lipopolysaccharide (LPS), lipid A and mannoproteins, are potential targets for AMPs. Specifically, Gram-positive and Gram-negative bacteria are classified based on their bacterial cell wall structure. Gram-positive bacteria have a layer of cross-linked peptidoglycan, whereas Gram-negative bacteria have an additional outer membrane with an inner leaflet containing only phosphatidic acid and an outer leaflet made of LPS. LPS has numerous negatively charged phosphate groups, which combine with a salt bridge with a divalent cation (e.g., Ca^2+^ and Mg^2+^) to form an electrostatic network (Nikaido, 2003). This electrostatic zone is the main barrier against hydrophobic antibiotics and causes the low permeability of Gram-negative bacteria. The main components of the fungal cell wall are mannoprotein, β-glucans and chitin (polymers of 1,4-β-*N*-acetylglucosamine) and the mutations in the relevant genes of the LPS pathway and phospholipid trafficking provide resistance to the AMPs (Cabib, 2009; Spohn et al., 2019). Mannoproteins in fungal cell walls include a variety of proteins, including structural proteins, cell adhesion proteins (floccrin and lectin) and enzymes involved in cell wall synthesis and remodeling (hydrolytic enzymes and transglycosylase). These proteins differ from human cell membrane proteins and are potential targets of AFPs (Rautenbach et al., 2016). Furthermore, teichoic acid and lipoteichoic acid in the cell wall are also potential targets of AMPs and these theories could support the design of AMPs with low cytotoxicity.

## Design Methods of Antimicrobial Peptides

Antimicrobial peptides have good application prospects. However, AMPs have the following problems. (1) AMPs damage the cell membrane of eukaryotes and cause hemolytic side effects; (2) rising production costs and technical problems limit their manufacture; (3) their stability is limited at certain pH; (4) AMPs have reduced activity under the presence of iron and certain serum; (5) AMPs are easily hydrolyzed by proteases. Therefore, the ideal AMP should meet the following characteristics: (i) high antimicrobial activity; (ii) low toxicity to mammalian membranes; (iii) high protease and environment stability; (iv) low serum binding capacity and (v) ease of access and low cost production (Li et al., 2017). Therefore, designing AMPs to achieve the desired effect has attracted increasing attention. The rational design of antibacterial peptides should focus on the following five aspects: chain length, secondary structure, net charge, hydrophobicity, and amphiphilicity and these have been mentioned in many studies and this review will focus more on several specific methods of antimicrobial peptide design.

### Site-Directed Mutation

Site-directed mutation refers to the redesign of natural antimicrobial peptides by adding, deleting or replacing one, or several amino acid residues (Torres et al., 2019).

### *De novo* Design Peptides

The *de novo* design of peptides attaches importance to the design of amphiphilic AMPs (Guha et al., 2019). For example, GALA is a well-known *de novo*-designed AMP. Amphipathic α-helical peptide GALA is created by placing protonatable glutamic acid residues in most positions with the spacing of i to *i* + 4 (Goormaghtigh et al., 1991). The repeated sequence (XXYY)n, where *X*_1_ and *X*_2_ are hydrophobic amino acids, *Y*_1_ and *Y*_2_ are cationic amino acids, and n is the number of repeat units, is designed based on the hydrophobicity cycle that mimics natural α-helical AMPs and successfully designs broad-spectrum α-helical AMPs. Sequences (LKKL)_3_ and (WKKW)_2__.__5_ have the highest selectivity (Khara et al., 2017). Moreover, L_l_K_m_W_2_ model peptides are also *de novo*-designed peptides. Amphipathic helical properties were conferred by using leucines and lysines, and two tryptophan residues were positioned at the amphipathic interface between the hydrophilic ending side and the hydrophobic starting side. Among the model peptides, L_4_K_5_W_2_ has good anti-MRSA activity (Lee et al., 2011).

### Template-Based Design Method

Sequence templates can be obtained by comparing a large number of structurally homologous fragments of natural AMPs (such as HDPs) and extracting conservative patterns based on the type of residue (such as charged, polar, hydrophobic, etc.) (Zelezetsky and Tossi, 2006). Based on the modification, the parameters, such as helix formation tendency, cationic, amphiphilicity and overall hydrophobicity, can be systematically changed. For instance, cecropin, magainin, protegrin, and lactoferrin have all been used as AMP templates (Fjell et al., 2012).

### Based on the Self-Assembly of Antimicrobial Peptides

Peptides can form nanostructures, such as micelles, vesicles, nanotubes, nanoparticle nanobelt, and nanofibre nanotube, and can increase or impart antibacterial activity to AMPs during the self-assembly of peptides. For example, KLD-12 (KLD) is a self-assembling peptide with 12 amino acid residues that can adopt nanostructures and are known for their tissue engineering properties. The addition of Arg residues in KLD shows no remarkable change in its β-sheet secondary structure and the self-assembly characteristics of the forming nanostructures (Tripathi et al., 2015). Dimer structure can also be used to enhance the antimicrobial activity of AMPs and reduce toxicity, but membrane-destabilizing effects are reduced after dimer formation (Malekkhaiat Häffner and Malmsten, 2018).

### Chemical Modification

Various chemical modifications of AMPs, including residue phosphorylation, the addition of D-amino acids or unnatural amino acids (homoarginine), cyclization, halogenation, acetylation, and peptidomimetics, have been used to improve the stability of peptides against proteases. Given that the enzyme is stereospecific, the incorporation of unnatural D-amino acids into the AMP sequence can reverse the stereochemistry and prevent protease degradation (Zhong et al., 2020). The so-called peptidomimetics, whose main elements mimic the structure of peptides, are usually produced by modifications, such as chain extension or heteroatom incorporation of existing peptides (Patch and Barron, 2002). Ornine, which is an unnatural residue with a positive charge and has a high resistance to protease activity, is also used in non-chemical modification. Replacing Trp residues with family residues, such as β-dihydrophenylalanine, can stabilize secondary structures and improve antibacterial properties (Maurya et al., 2013).

#### Halogenation

Halogenation is highly related to the activity, specificity, and stability of AMPs. In the latest report, Halogen is introduced into jelleine-I which is a short peptide isolated from the royal jelly of honeybees (*Apis mellifera*) by replacing phenylalanine with a halogenated phenylalanine analog, increasing the antibacterial activity *in vitro* and anti-biofilm activity. In addition, the proteolytic stability of jelleine-1 is increased by 10–100 times by halogenation (Jia et al., 2019). The halogenated peptidomimetic α,α-disubstituted β-amino amides are also promising bacteriostatic drugs that have inhibitory effects on more than 30 multi-resistant clinical isolates of Gram-positive and Gram-negative bacteria (Paulsen et al., 2019). Halogenation is also related to the specificity of AMPs. The o-fluorine substitution in phenylalanine residues maintains the activity of temporin L on *E. coli* but leads to the loss of activity on *S. aureus* and *P. aeruginosa* (Setty et al., 2017).

#### Cyclisation

Three modes of cyclisation, including cyclisation via disulfide bonds, head-to-tail cyclisation and internal bonding between side chains, have been found in natural AMPs. The synthesis of disulfide bonds often complicates the development of synthetic peptides. The circularisation of the main chain of arenicin-1 molecule resulted in increased activity against drug-resistant clinical isolates but caused no substantial effect on cytotoxicity (Orlov et al., 2019). The HDPs tachyplesins I, II, and III and their cyclic analogs cTI, cTII, and cTIII, respectively, have similar structures and activities and can resist bacterial and cancer cells. The cyclisation of the backbone reduces the hemolytic activity and improves the stability of the peptides whilst maintaining effective anticancer and antibacterial activities (Vernen et al., 2019).

#### Capping

Capping refers to the addition of specific motifs or modifications, such as amidation at the C-terminus and acetylation at the N-terminus, rendering AMPs with more natural peptide characteristics. Post-translational modifications play an important role in the function of AMPs and are the most commonly used in peptide design. The C-terminal Rana box (consisting of a C-terminal cyclic heptapeptide with a conservative disulfide bond) and amide group are important C-terminal capping methods. For example, the C-terminal amide group of maximin H5 can enhance antibacterial efficacy without increasing lytic ability (Dennison et al., 2015). The N-terminal lipidated analog C_4_VG_16_KRKP shows enhanced antibacterial activity against various Gram-negative bacteria. The functions of N-terminal lipidation include (i) increasing LPS neutralization, (ii) increasing stability to proteases and peptidases, and (iii) reducing cytotoxicity (Datta et al., 2016). Furthermore, hydrophobic end labeling is a commonly used method to increase the activity of antimicrobial peptides. Acyl lipid peptides have a linear or cyclic structure in which one or more hydrocarbon tails are connected to the N-terminus of a short oligopeptide (Chu-Kung et al., 2010). Lipopeptides have covalently attached hydrophobic moieties, such as sterols or fatty acids. Aromatic amino acid terminal labeling is also the main hydrophobic terminal labeling method. Tryptophan (W) and phenylalanine (F) are the commonly used aromatic amino acids. Their large and polarisable residues have an affinity for the interface, and the W/F tag is also sensitive to the differences between ergosterol and cholesterol and can prevent self-assembly. This condition results in low aggregation numbers and high critical aggregation concentrations (Schmidtchen et al., 2014).

#### Conjugation

Peptide conjugation has been the goal of most research in recent years to produce active and stable AMPs with high selectivity. Different side chains or AMP fragments can be used aside from the repetition of the same amino acid motifs. For example, conjugating fatty acids with a length of 8–12 carbon atoms to the 4th or 7th side chain of the D-amino acids of Ano-D_4_,_7_ improves antibacterial selectivity and anti-biofilm activity. In addition, the new peptide exhibits high stability against trypsin, serum, salt, and different pH environments (Zhong et al., 2020). The conjugation of different AMPs can also be performed. For example, the hybrid peptide (PA2–GNU7) constructed by the addition of PA2 to GNU7 has a high activity and specificity to *P. aeruginosa* (Kim et al., 2020).

#### Synthetic Mimics of AMPs (SMAMPs)

SMAMPs include a broad family of molecular entities based on the structure and function of AMPs. However, their backbones are not entirely based on α-amino acids, including β-amino acid oligomers, arylamide oligomers, and phenylene ethynylenes (Michael Henderson and Lee, 2013). For instance, SMAMP10, which is a potential drug for intravenous treatment, causes no drug resistance and has a strong inhibitory effect on *MRSA* and vancomycin-resistant *Enterococcus faecium* (Tew et al., 2006).

##### Peptoids

Peptoids are peptide isomers, in which the side chain is bonded to the main chain nitrogen instead of α-carbon or poly-N-substituted glycine in which the side chain is connected to amide nitrogen instead of the α-carbon on the main chain (Andreev et al., 2018). For example, the cationic peptide SA4 (IOWAGOLFOLFO-NH2) and its poly-N-substituted glycine homolog SPO (nInOnWnAnGnOnLnFnOnLnFnO-NH_2_) inhibit the planktonic and biofilm formation of *A. baumannii* strains, which are susceptible to multi-drug resistance (Sharma et al., 2019).

### Use of Motifs

Motifs with specific functions have been reported increasingly. These motifs can be repeated units for combining into new antimicrobial peptides, or specific amino acid combination units appearing at the end (such as capping) of or even in the peptide chain.

#### Motif at the End of the Chain

##### ATCUN motif

This motif includes two tripeptide structures, including Gly–Gly–His or Val–Ile–His, which are added at the end of the peptide chain. ATCUN-containing AMPs in the presence of hydrogen peroxide and ascorbic acid combine with Cu^2+^ to induce the valence of copper ions between +2 and +3 oxidation states and form an ATCUN–Cu (II) complex, generating ROS by Fenton-like reactions. Extracellular polymeric substances (EPS) are important for biofilms and can enhance the resistance of cells to antibacterial agents (Flemming, 2016). ATCUN–AMPs have been used to degrade environmental DNA, which is one of the major components of EPS. Several related practical applications have been reported. For example, the biological activity against carbapenem-resistant Enterobacteriaceae is increased by adding this motif to the N-terminus of an alpha-helical AMP (such as CM15). Besides, the Cu–ATCUN derivative of OV-3 containing a C-terminal GGC sequence showed high levels of membrane permeation and lipid peroxidation. The concept of catalytic metal drugs has attracted widespread attention although the concept is still in its infancy because of the role of metal ions (Alexander et al., 2017; Agbale et al., 2019).

##### Rana box

Rana box: Rana box is a heptapeptide motif (CGLXGLC) from the nigrocin family. Rana box consists of two cysteine residues that are separated by four or five other residues on the side and can form a cyclic disulfide bond. Rana box peptide has shown structural analogies with polymyxin (colistin), and the primary structure of the Rana box motif is important in determining bacteriostatic activity (Kozić et al., 2015). The deletion of the ‘Rana box’ motif will cause the AMP antibacterial effect to disappear, but replacing the natural ‘Rana box’ sequence of AMPs with amidated phenylalanine can expand its efficacy against antibiotic-resistant microorganisms, including *MRSA* and *P. aeruginosa*, and reduce cytotoxicity. This phenomenon also shows that the effect of the motif on AMPs needs to be determined based on the specific situation and is not completely beneficial (Bao et al., 2018).

##### LPS binding motif

The LPS binding motif (G-WKRKRF-G) can produce a broad spectrum of antibacterial activity when introduced into the C-terminus of temporin-1 Ta and temporin-1 Tb (close isoforms of temporin) (Mohanram and Bhattacharjya, 2016).

##### γ-core motif

Antifungal Peptides have a conserved GXC(X_3__–__9_) C γ-core motif (residues 5–14, GKCYKKDNIC; d-isomer) at its N-terminus, which is a cation part of the ring. This conserved motif interferes with the integrity of the plasma membrane of the cell (Yount and Yeaman, 2004). Conserved γ-core motifs are directly involved in protein–membrane interactions and strongly contribute to membrane binding (Utesch et al., 2018).

#### Motif in the Chain

If replace d-Phe1-Pro2 sequence in peptide chain with d-Phe-2-Abz turn motif (2-Abz is an abbreviation of 2-aminobenzoic acid D-amino acid) in AMP Tyrc A, and nuclear magnetic resonance shows that this change retains the β-hairpin structure. Unlike the traditional β-turn motif, the D-Phe-2-Abz motif can be used as a tool for β-hairpin libraries. The hydrophobic peptide can be formed into the nucleated β-hairpin formation by adding the D-Phe-2-Abz motif. Moreover, the inclusion of this part in two designed cationic amphiphilic peptides can produce broad-spectrum antibacterial activity and low hemolysis rate (Cameron et al., 2017; Cameron et al., 2018).

##### NGR motif

The NGR motif is composed of Asn–Gly–Arg, and AMPs with this structure have strong cytotoxicity (Table 1). The data indicate that the new AMPs containing NGR may bind to CD13+ or αvβ3+ tumor cells by binding to CD13 or αvβ3, respectively, to exert anti-tumor activity, especially on CD13+ tumor cells (Zhang et al., 2015).

**TABLE 1:** Examples of AMPs with NGR motif.

Name	Sequence	3D structure	Activity	Reference
CORTICOSTATIN I	ICACRRRFCPNSERFSGY CRVNGARYVRCCSRR	Bridge	Anti-Gram+ and Gram−	Zhu et al., 1988
NP-3a	GICACRRRFCPNSERFSGY CRVNGARYVRCCSRR	Bridge	Anti-Gram+ and Gram−, Antiviral, and Antifungal	Selsted et al., 1985
Corticostatin VI	GICACRRRFCLNFEQFSG YCRVNGARYVRCCSRR	Bridge	Anti-Gram+ and Gram−	Fuse et al., 1993
Pediocin PA-1/AcH	KYYGNGVTCGKHSCSVDWGKA TTCIINNGAMAWATGGHQGNHKC	Combine Helix and Beta structure	Anti-Gram+, Spermicidal	Henderson et al., 1992
Lacticin 3147	CSTNTFSLSDYWGNNGA WCTLTHECMAWCK	Helix	Anti-Gram+, Spermicidal	Martin et al., 2004
As-CATH5	TRRKFWKKVLNGALKIAPFLLG	Helix	Anti-Gram+ and Gram−, Antifungal, anti-sepsis	Chen Y. et al., 2017

##### “Glycine zipper” of GxxxG motifs

The central GxxxG motif can induce strong self-assembly and have been already used in the design of AMPs (Brosig and Langosch, 1998; Krauson et al., 2012).

#### Motif-Based Polyvalent Peptide Synthesis (Dimers, Tetramers, etc.)

Bovine lactoferrin B is an AMP composed of 25 amino acid residues and has antibacterial, antifungal, and antiparasitic activities. The multivalent molecules LfcinB (20–25)_2_ and LfcinB (20–25)_4_ contain the LfcinB (20–25) motif (RRWQWR) and show inhibition activity against *E. coli*, *P. aeruginosa*, and *S. aureus*. Chimeric peptide chimera 3 containing two motifs, namely, the RRWQWR of LfcinB (20–25) and the RLLR of BFII (32–35), shows high antibacterial activity against *E. coli* ATCC 25922 and *S. aureus* ATCC 25923 (Vargas-Casanova et al., 2019; Pineda-Castañeda et al., 2020).

### Computer Design

Computer design includes simple statistical modeling, Structure-activity relationships study (Abdel Monaim et al., 2018), neural networks (Müller et al., 2018), deep learning (Veltri et al., 2018), word embedding (Hamid and Friedberg, 2018) and machine learning. For example, a machine learning method by Matlab is proposed based on the concept of scoring the contribution of each amino acid’s antibacterial activity (Wu X. et al., 2014). The genetic algorithm was used to design the amphiphilic α-helical peptide guavalin 2, which has an uncommon amino acid composition (three tyrosine and three glutamine residues) and interestingly causes membrane hyperpolarization, which is a different mechanism from those of other AMPs (Porto et al., 2018). Two research methods have been developed based on the research background of quantitative structure–activity relationships: prediction method based on AMP therapeutic index and the identification of novel potential AMPs from the expressed sequence tag database based on the principles of the highly conserved signal peptide subclasses related to AMPs (Juretić et al., 2011).

### Rational Library Design

In this way, a variety of AMP variants can be obtained. If combined with high-throughput screening, it can effectively obtain the desired AMP. For instance, some new AMPs are designed by the combinatorial peptide library of melittin and show higher activity and lower cytotoxicity (Krauson et al., 2015).

## Environmental Factors Affecting the Activity of Antimicrobial Peptides

### Metal Ions

Cations, such as Na^+^ and Mg^2+^, may affect AMP activity (Zhu et al., 2015). However, the different valences of metal ions have varied effects on AMPs. For example, divalent cations show stronger antagonism to bacteria than monovalent cations with thanatin and s-thanatin, which are insect AMPs (Wu et al., 2008). In the presence of NaCl, the signal response during the association phase remarkably decreased in single-cycle and multi-cycle kinetic experiments, resulting in a decreased association rate. This occurrence may be caused by the shielding effect of NaCl between the cationic peptide and the zwitterionic membrane. Another possible reason is that Na^+^ can bind to the phospholipid bilayer, where the ions interact with the phosphate and the carbonyl oxygen of lipid head groups (Sabapathy et al., 2020). The reduced activity of synthetic peptide [RLLR]_5_ under high salt concentration is possibly caused by the destruction of its α-helix structure.

Table 2 shows that several AMPs, including histatin, myxinidin, and hepcidin, contain ATCUN motifs (Amino Terminal Copper and Nickel with XXH sequence). Iron is the most abundant metal ion in human saliva, but the combination with this metal ion results in the loss of the α-helix of histatin 5 and greatly reduces its antifungal activity (Puri et al., 2015). However, the coordination of copper (II) and nickel (II) ions can induce the formation of ROS, which is essential for bactericidal activity (Jeżowska-Bojczuk and Stokowa-Sołtys, 2018).

**TABLE 2:** Effect of metal ions on AMPs with ATCUN motif activity.

Name	Source	Sequence	Details
Histatin 5	Human parotid saliva	DSHAKRHHGYKR KFHEKHHSHRGY	It losses the α-helical structure by binding iron and coordination of copper (II) and nickel (II) ions induces the ROS.
Myxinidin	Epidermal mucus of hagfish	GIHDILKYGKPS	It is similar to that of other peptides with the ATCUN motif.
Hepcidin 25	Human liver	DTHFPICIFCCGC CHRSKCGMCCKT	When in the presence of copper (II) ions and an intracellular substance such as ascorbate, hepcidin 25 may generate ROS.

Anionic AMPs have a large number of negatively charged aspartic and glutamic acid residues (Lakshmaiah Narayana and Chen, 2015). They require zinc as a functional cofactor and the zinc complex shows stronger antibacterial activity (Jiang et al., 2014). Several of these AMPs use metal ions to form cationic salt bridges with the negatively charged components of the microbial membrane to penetrate the membrane. Anionic AMPs may attach to ribosomes or inhibit ribonuclease activity when in the cytoplasm (Jeżowska-Bojczuk and Stokowa-Sołtys, 2018).

Metal ions also affect the self-assembly of peptides. These ions can recognize specific amino acids, such as lysine and glutamic acid, and may form salt bridges between peptide molecules to induce peptide self-assembly. For example, Zn^2+^ can stabilize the aggregation of peptides on the cell membrane, which results in the enhanced antibacterial effect of DCD-1L in the presence of Zn^2+^ (Tian et al., 2015).

### pH

Many AMPs are stable and retain their antimicrobial activity in a wide pH range. AMPs have enhanced activity at low pH because of their basic properties. This condition is related to the protonation of histidine at acidic pH, which promotes electrostatic interactions with anionic surfaces, including LPS and the anions of phospholipids, and subsequently enhances antibacterial properties. The effect of pH on the antibacterial activity of AMPs varies. For example, thanatin’s activity at neutral pH is slightly higher than that under acidic conditions. By contrast, the activity of xylan on *E. coli*, *Listeria*, and *C. albicans* is remarkably higher at pH 5.5 than at pH 7.4 (Holdbrook et al., 2018). The inactivation of the histidine-containing AMP C18G-His under low pH conditions involves pH-dependent changes in the state of the aggregates in the solution, because the aggregates, which are sensitive to pH and lipid composition, may be affected by binding and conformation. Peptides can also enhance bacterial membrane permeability at low pH (Hitchner et al., 2019). Thrombin-derived C-terminal peptides (TCPs) will also change the mode of CD14 (a protein that is abundant in human plasma) from anti-inflammatory mode to bacterial elimination mode from pH 7.4 to pH 5.5 (Holdbrook et al., 2018). A dimer (e.g., P-113) can be created to provide AMPs with resistance to a higher pH range. The sensitivity of this pH-sensitive AMP can be used to achieve a certain targeting effect in practical applications. In addition, charge interaction is one of the most important factors in peptide self-assembly. pH affects the charge state of amino acid and substituent functional groups. Therefore, adjusting the pH is the most common method for controlling peptide assembly and disassembly (Tian et al., 2015).

### Proteases

Proteases have a strong destructive effect on AMPs. For instance, LL-37, which has the strongest inhibitory effect on chlamydial infection, is inhibited by the protease chlamydial *protease*-like activity factor (CPAF) secreted by *Chlamydia* (Tang et al., 2015). Studies have been focused on the design of AMP carriers to solve this problem (Lewies et al., 2017; Nordström et al., 2019). The presence of chitosan–silica solid support of KR-12 peptide can protect it be hydrolyzed by α-trypsin, and the degree of protection is increased by 38% compared with the free KR-12 (Diosa et al., 2020). However, several enzymes, such as protease 65, esterase 66 and phosphatase 67, cut the blocking group of the peptide and trigger the self-assembly of the peptide, which positively affects AMPs (Tian et al., 2015).

## Current Progress and Application of Antimicrobial Peptides

### Medicine

Antimicrobial peptides can regulate pro-inflammatory reactions, recruit cells, stimulate the proliferation of cells, promote wound healing, modify gene expression and kill cancer cells to participate in the immune regulation of human skin, respiratory infections, and inflammatory diseases (de la Fuente-Núñez et al., 2017). For example, α-defensins HNP-1, HNP-2, and HNP-3 showed effective antibacterial activity against adenovirus, human papilloma virus, herpes virus, influenza virus and cytomegalovirus. Pulmonary diseases, such as idiopathic pulmonary fibrosis, alveolar proteinosis, and acute respiratory distress syndrome, show elevated levels of AMPs (Guaní-Guerra et al., 2010). Likewise, AMPs secreted by the Paneth cells in the mammalian gut are important to shape the gut microbiota (Bevins and Salzman, 2011).

The application of AMPs in medicine, such as dental, surgical infection, wound healing and ophthalmology is developing now. But there are only three AMPs that have been approved by FDA including gramicidin, daptomycin, and colistin.

Dental caries, endodontic infections, candidiasis, and periodontal disease are common diseases in the human oral cavity. Dental caries is a prevalent oral disease and some acidogenic bacteria like *Streptococcus* sp. are the main caries-associated pathogens (Izadi et al., 2020). Several AMPs have good application potential. For instance, peptide ZXR-2 (FKIGGFIKKLWRSLLA) has shown potent activities against pathogenic bacteria of dental caries, *Streptococcus mutans*, *Streptococcus sobrinus*, and *Porphyromonas gingivalis* and peptide PAC-113 (Clinical trial identifier: NCT00659971) that has been sold over the counter in Taiwan for treating oral candidiasis (Chen L. et al., 2017).

In surgical infection and wound healing: surgical infection occurs after surgery, burns, accidental injury, skin disease, and chronic wound infections have a serious hazard to human life (Thapa et al., 2020). Several AMPs have shown the therapeutic potential of these diseases. For example, AMP PXL150 shows pronounced efficacy as an anti-infective agent in burn wounds in mice and AMP D2A21 has been in the third phase of clinical trials for treating burn wound infections (Björn et al., 2015).

In ophthalmology: Human eyes are prone to be infected by several organisms including bacteria and fungi in which *S. aureus*, *Streptococcus pneumoniae*, *P. aeruginosa*, *Aspergillus* spp., and *C. albicans* are the most relevant pathogens (Silva et al., 2013). Although AMPs such as Lactoferricin B, Protegrin-1 exhibited antimicrobial activity against these pathogenic bacteria, their application in the field of ophthalmology is only at the theoretical stage. With the popularity of contact lenses and the increase in cases of related eye infections, antimicrobial peptides have shown good application prospects in ophthalmology (Khan and Lee, 2020).

Additional methods need to be performed for the application of AMPs as drugs in medicine. The main strategies include (1) constructing precursors to reduce cytotoxicity and improve protease stability, (2) using AMPs in combination with existing antibacterial agents, (3) inducing the correct expression of AMPs with appropriate drugs and using engineering probiotics as vectors to express AMPs. For example, in the field of wound repair, different formulation strategies, such as loading AMPs in nanoparticles, hydrogels, creams, gels, ointments, or glutinous rice paper capsules, have been developed to effectively deliver AMPs to the wound (Borro et al., 2020; Thapa et al., 2020). In recent research, the sponges developed from modified starch and HS-PEG-SH are covalently immobilized with AMP showed effective antibacterial activity (Yang et al., 2019).

More technical means, including pheromone-labeled AMPs, local environment-triggered AMPs (enzyme precursor drug release system, pH-activated AMPs, etc.), have been developed to improve the targeting mechanism of AMPs. Furthermore, nanotubes, quantum dots, graphene, and metal nanoparticles have been proposed to be a potential method to enhance drug delivery of AMPs (Magana et al., 2020). Hybrid peptides have also been used to build targeting peptides. For example, PA2, which is a *P. aeruginosa*-targeting peptide, was combined with GNU7 (a broad-spectrum AMP) to construct a hybrid peptide (PA2–GNU7) that targets OprF protein and has good bactericidal activity and specificity (Kim et al., 2020). Furthermore, some antibiotics, for instance, daptomycin (a lipopeptide), lugdunin which is a 21-membered cyclic peptide consists of 6 amino acid residues plus a thiazolidine moiety and telavancin (a glycopeptide) have been widely used for the clinic (Durand et al., 2019; Lampejo, 2020). Although they are antibiotics, they have provided broader ideas for the design of AMPs.

### Food

Food preservatives have potential harm to the human body. Therefore, natural preservatives are being advocated by more people. AMPs have a good inhibitory effect on common bacteria and fungi in food, and many AMPs are resistant to acids, alkalis, and high temperatures are easily hydrolyzed by proteases in the human body. Thus, AMPs are a promising alternative to preservatives. Nisin is a bacteriocin produced by *L. lactis* subspecies. Lactic acid bacteria have been widely used as food preservatives. Nisin is categorized as generally recognized as safe (GRAS) by the US Food and Drug Administration (FDA) and is used as a food preservative in other countries (Khan and Oh, 2016). However, only nisin and polylysine are currently approved by the FDA as food additives (Santos et al., 2018). Pedocin PA-1, a bacteriocin consisting of 44 amino acids produced by a diplococcus, is also used as a food preservative and is sold on the market under the trade name ALTA 2431. Pedocin PA-1 is used as a food additive to inhibit the growth of *L. monocytogenes*, which can cause meat deterioration (Settanni and Corsetti, 2008). Enterocin AS-48 is an AMP used to preserve cider, fruit and vegetable juices, and enterocin CCM4231 is used to preserve soy milk (Rai et al., 2016; Santos et al., 2018). Encapsulating bacteriocins into liposomes is a new method used to overcome the problems of AMPs in food applications (such as proteolytic degradation or interaction with food ingredients) (da Silva Malheiros et al., 2010).

Moreover, active packaging by adding AMPs is a novel packaging method that has great potential in the food industry. For instance, ε-poly-L-lysine is used in conjunction with starch biofilms to show good inhibitory effects on *Aspergillus parasiticus* (aflatoxin producer) and *Penicillium expansum* and nisin have the potential to be dairy preservative because it is a highly surface-active molecule (Luz et al., 2018).

### Animal Husbandry and Aquaculture

The European Union banned the use of animal growth promoters in animal feed in 2006. Thus, a new antibacterial strategy is needed. Many AMPs are the potential to be used in poultry, swine, and ruminants breeding and aquaculture because they can improve production performance (Liu et al., 2008; Bao et al., 2009), immunity and promote intestinal health and some of them have a stronger inhibitory effect on bacterial inflammation if used with antibiotics (Wang et al., 2019; Cote et al., 2020). For example, SIAMP has a good effect on the treatment of IBV in chicken (Sun et al., 2010). By adding swine gut intestinal antimicrobial peptides (SGAMP), broilers showed higher average daily gain and feed efficiency under chronic heat stress conditions (Hu et al., 2017). Frog caerin 1.1, European sea bass dicentracin and NK-lysine peptides (NKLPs) have good inhibitory effects on *Nodavirus*, *Septicaemia haemorrhagic virus*, *Infectious pancreatic necrosis virus* and *Spring viremia carp virus*, which are devastating to fish farming (León et al., 2020). The AMP in soybean meal fermented by *B. subtilis* E20 effectively inhibits *V. parahaemolyticus* and *Vibrio alginolyticus* and enhances the resistance level of *Litopenaeus vannamei* against *V. parahaemolyticus* when added to feeds (Cheng et al., 2017).

### Agriculture

For agriculture, the plant pathogenic infection of bacteria and fungi causes the loss of economy, for instance, *Aspergillus flavus* infection of corn and peanuts, citrus green mold caused by *Penicillium digitatum*, gray mold disease caused by *Botrytis cinerea* on strawberries and Geotrichum *citri-aurantii* infection of citrus fruit all cause great harm to the growth and post-harvest of agricultural products (Liu et al., 2007; Liu et al., 2019). Several AFPs have shown prospect to control these problems. However, the practical application of antimicrobial peptides in the transportation and preservation of agricultural products is still lacking, because the use of antimicrobial peptides will greatly increase the cost in the transportation of fruits and vegetables (application examples of AMPs in these four fields are shown in Table 3).

**TABLE 3:** Application examples of AMPs in various fields.

	AMPs/Product name	Description	Treatment/effect	Company/reference
**Medicine**	Dalbavancin (BI397, Dalvance, Xydalba)	Semisynthetic lipoglycopeptide	Acute bacterial skin infections	Approved
	PAC-113, P-113	Histatin 5 derivative (12 amino acids)	Oral candidiasis	Phase IIb complete (sold over the counter in Taiwan by General Biologicals Corporation)
	Fuzeon	Enfuvirtide	HIV-1 infection	Approved
	Baciim	Bacitracin	Localized skin and eye infections, wound infections	Approved
	Vancocin	Vancomycin	Bacterial infections	Approved
	Daptomycin	Lipopeptide	Gram-positive infections	Approved
	Telavancin	Glycopeptide (lipoglycopeptide)	Skin infections, osocomial pneumonia	Approved
	Colistin	Polymyxin E	MDR infections caused by Gram-negative bacteria	Approved
	Gramicidin	Cationic cyclic deca-peptide	Purulent skin disease	Approved
	D2A21	Synthetic peptide	Burn wound infections	Phase III/Demegen
	PXL01	Lactoferrin analog	Postsurgical adhesions	Phase III/ProMore Pharma
	Omiganan (CLS001)		Papulopustular rosacea;	Phase III
**Food**	Nisin		Dairy (*Listeria monocytogenes* and *Staphylococcus aureus*)	Approved
	Polylisine	Natural cationic antibacterial agent	Sushi, boiled rice, noodles, meat, and drinks	Approved
**Husbandry**	SGAMP	Swine gut intestinal antimicrobial peptides	Heat stress	Hu et al., 2017
	NKL-24	Zebrafish NK-lysin	*V. parahaemolyticus* infection in the scallop	Shan et al., 2020
	Caerin1.1	Magnificent tree frog	*L. garvieae*, porcine epidemic diarrhea virus (PEDV)	León et al., 2020
	Dicentracin	European sea bass	*L. garvieae*, viral hemorrhagic septicaemia (VHSV), infectious pancreatic virus (IPNV)	León et al., 2020
**Agriculture**	PAF26	RKKWFW	Green mold	Wang et al., 2018
	O3TR/C12O3TR	H-OOWW-NH2/C_12_-OOWW-NH2	*P. digitatum*	Li X. et al., 2019
	Thanatin	*Podisus maculiventris* (GSKKPVPIIYCNRRTGKCQRM)	Rice blast disease, Sour rot (Geotrichum *citri-aurantii*)	Imamura et al., 2010
	Ponericin W1	*Pachycondyla goeldii* (WLGSALKIGAKLLPSVVGLFKKKKQ)	*M. oryzae*, *Botrytis cinerea*, and *Fusarium graminearum*, Sour rot (Geotrichum *citri-aurantii*)	Orivel et al., 2001
	Mastoparan-S	*Sphodromantis viridis* (LRLKSIVSYAKKVL)	G+, G−, *Aspergillus niger*, *Aspergillus fumigates*, Sour rot (Geotrichum *citri-aurantii*)	Zare-Zardini et al., 2015

## Conclusion

Antimicrobial peptides constitute a global research hotspot, but many key issues in design and application need to be solved urgently. Several restrictive factors hinder the application of AMPs. The interaction of multidisciplinary subjects, such as biology, materials science, chemistry, bioinformatics, molecular informatics and pharmacy can further develop prospective AMPs. Computer molecular dynamics simulation, cell membrane simulation, and more methods are being applied to study the mechanism of AMPs. How to further understand the correlation between AMPs and various targets instead of conducting one-sided experimental research might improve experimental designs to obtain stronger systemic and scientific demonstrations. On this basis, further animal experiments are required instead of simple cell-level experiments to test the effect of AMPs under complex physiological conditions. Several complicated methods, such as the chemical method of peptidomimetics and non-natural amino acid modifications, have been applied in designing AMPs to solve the problem of protease hydrolysis. Most methods use chemical substrates, but the cost of these methods cannot be ignored in practice. In addition, chemical synthesis and the use of engineered bacteria are currently the mainstream for such procedures. Finding a better biological preparation method, reducing the cost and increasing the yield is important problems in practical application. Furthermore, studying the AMP expression of the organism itself and finding a better expression vector are necessary for mass production in the future as more AMPs in nature are discovered. Further research is needed on the reported AMPs to solve the problem on structure–function relationship. As a branch of peptide drugs, AMPs need to progress with the advancement of medical science against the background of the current low success rate of the clinical application of AMPs. More attention can be focused on food, agriculture, and animal husbandry.

## Author Contributions

QK and YH: conceptualization, methodology, writing – original draft preparation, and writing – review and editing. All authors contributed to writing and reviewing the manuscript.

## Conflict of Interest

The authors declare that the research was conducted in the absence of any commercial or financial relationships that could be construed as a potential conflict of interest.

## Footnotes

1 http://aps.unmc.edu/AP/

2 http://crdd.osdd.net/servers/avpdb/
